# Supplementary material for: EnzML: multi-label prediction of enzyme classes using InterPro signatures
Source: BMC Bioinformatics. 2012 Apr 25;13:61. doi: 10.1186/1471-2105-13-61 (PMC3483700; doi:10.1186/1471-2105-13-61)
Supplement: Addtional file 5 — The Java code to format the data files, evaluate and predict. The file enzml_java_code.tar.gz contains the Java code used to format database data to ARFF and XML formats, to execute cross and train-test (jackknife) evaluations and to record evaluation results to database. More information is included in the readme.txt file and the Javadoc files. The code can be used with a MySQL database. To use a different database software, other JDBC drivers might be required. [file 1471-2105-13-61-S5.gz › java_code/utils/doc/uk/ac/ed/inf/utils/ReflectionUtils.html]

ReflectionUtils


---


|  |  |  |
| --- | --- | --- |
| **Overview** \| **Package** \| **Class** \| **Use** \| **Tree** \| **Deprecated** \| **Index** \| **Help** | |  |
| **PREV CLASS**   **NEXT CLASS** | **FRAMES**    **NO FRAMES**     **All Classes** |  |
| SUMMARY: NESTED \| FIELD \| CONSTR \| METHOD | DETAIL: FIELD \| CONSTR \| METHOD |  |


---


## uk.ac.ed.inf.utils Class ReflectionUtils

```
java.lang.Object
  uk.ac.ed.inf.utils.ReflectionUtils
```

---

``` public class ReflectionUtils extends java.lang.Object ```

Class

**Version:**
:   24 Feb 2011

**Author:**
:   Luna De Ferrari luna.deferrari-at-ed.ac.uk

---

**Constructor Summary**

`ReflectionUtils()`


| **Method Summary** | |
| --- | --- |
| `static java.lang.reflect.Field[]` | `classFieldNames(java.lang.Object o)`             public static String classFieldsNamesAndValuesString(Object o){ TreeMap map = ReflectionUtils.classFieldsNamesAndValues(o); String fieldsValues = "Fields of " + o.getClass().getName() + "\n"; Set> entries = map.entrySet(); Iterator> it = entries.iterator(); while(it.hasNext()){ Entry entry = it.next(); fieldsValues += entry.getKey() } return fieldsValues; } |
| `static java.util.TreeMap<java.lang.String,java.lang.String>` | `classFieldsNamesAndValues(java.lang.Object o)` |

**Methods inherited from class java.lang.Object**

`equals, getClass, hashCode, notify, notifyAll, toString, wait, wait, wait`

**Constructor Detail**

### ReflectionUtils

```
public ReflectionUtils()
```


**Method Detail**

### classFieldNames

```
public static java.lang.reflect.Field[] classFieldNames(java.lang.Object o)
```

:   public static String classFieldsNamesAndValuesString(Object o){
    TreeMap map =
    ReflectionUtils.classFieldsNamesAndValues(o); String fieldsValues =
    "Fields of " + o.getClass().getName() + "\n"; Set>
    entries = map.entrySet(); Iterator> it =
    entries.iterator(); while(it.hasNext()){ Entry entry =
    it.next(); fieldsValues += entry.getKey() }
    return fieldsValues;
    }

---


### classFieldsNamesAndValues

```
public static java.util.TreeMap<java.lang.String,java.lang.String> classFieldsNamesAndValues(java.lang.Object o)
```


---


|  |  |  |
| --- | --- | --- |
| **Overview** \| **Package** \| **Class** \| **Use** \| **Tree** \| **Deprecated** \| **Index** \| **Help** | |  |
| **PREV CLASS**   **NEXT CLASS** | **FRAMES**    **NO FRAMES**     **All Classes** |  |
| SUMMARY: NESTED \| FIELD \| CONSTR \| METHOD | DETAIL: FIELD \| CONSTR \| METHOD |  |


---
